# Supplementary material for: Patient perceived weight stigma and patient-centered language use preferences: A cross-sectional mixed methods analysis conducted in a large academic medical center
Source: PLoS One. 2025 Feb 10;20(2):e0314269. doi: 10.1371/journal.pone.0314269 (PMC11809864; doi:10.1371/journal.pone.0314269)
Supplement: S1 File — (DOCX) [file pone.0314269.s001.docx]

**S1 File. NRC OHSU obesity guidelines survey**

# Community Insights Research: Obesity Guidelines, OHSU

### **Project Scope**

Learn and understand from our patients how they wish to engage with their providers when engaging in discussions surrounding weight. Learn preferences about the timing those discussions should occur, if they have experienced weight bias, and how patients would like to engage further with their providers regarding weight.

**EMAIL INVITATION DETAILS**

**Customer Logo:** OHSU logo was included

**Sender Name:** OHSU

**Sender Email:** OHSU@surveys.nationalresearch.com

**Subject Line:** Request for Feedback

**Email Body:** Your opinion is important to us! As a member of our community this is your chance to make a difference in how we provide healthcare information.

You can help us improve by taking this 5-minute survey about speaking to healthcare professionals. Please be honest, as we take your suggestions to heart.

Please follow the link below to access the survey.

[“Give us your feedback” link]

As a reminder, you are receiving this email because you have opted-in as a member of OHSU’s Community Insights panel. As a member, you will be invited to participate in surveys to provide feedback on various topics and influence decisions at OHSU. Your feedback is not only appreciated, but it is invaluable in helping OHSU achieve its mission.

Sincerely,

OHSU

**SURVEY INTRODUCTION**

Welcome to our survey! The following questions will ask about your experiences with and perceptions of healthcare.

**CORE QUESTIONS**

1. A primary care provider is a doctor, nurse practitioner, or physician assistant who sees patients for routine appointments and common medical conditions. A primary care provider is also known as a general practitioner or family doctor.

Do you have primary care provider you visit regularly?

- Yes, I have a primary care provider with OHSU
- Yes, I have a primary care provider somewhere else
- No
- Unsure

1. Weight management is what we call the efforts people make to gain, lose, or maintain a certain weight. Weight management often includes healthy eating and daily physical activity.

Has a primary care provider ever talked with you about weight management, or said that you are overweight or obese during an appointment?

- Yes
- No
- I prefer not to answer

1. [if ‘no’ in Q2] Would you have preferred your primary care provider to have spoken to you about weight management?
   - Yes
   - No
   - Unsure
2. [If ‘yes’ in Q2] Which of the following topics did you discuss with your primary care provider? [rotate responses, multi-select]
   - Physical activity (exercise) patterns
   - Eating patterns (including times of eating too little or too much)
   - Social factors that relate to eating and activity
   - Other, please specify: [open-ended, anchor]
3. [If ‘yes in Q2] Overall, how comfortable were you with the healthcare provider practitioner bringing up weight during your healthcare appointment?
   - Very comfortable
   - Somewhat comfortable
   - Neither comfortable nor uncomfortable
   - Somewhat uncomfortable
   - Very uncomfortable
4. [if ‘somewhat uncomfortable’ or ‘very uncomfortable’ in Q5] In your own words, what made you uncomfortable when the healthcare provider brought up weight management? [essay, optional]
5. Overall, how comfortable are you bringing up your weight to your healthcare provider?
   - Very comfortable
   - Somewhat comfortable
   - Neither comfortable nor uncomfortable
   - Somewhat uncomfortable
   - Very uncomfortable
6. [if ‘somewhat uncomfortable’ or ‘very uncomfortable’ in Q7] In your own words, why do you get uncomfortable bringing up your weight to your healthcare provider? [essay, optional]
7. Imagine you or a loved one could benefit from weight management. How would you want a healthcare provider to talk to you about weight management? [multi-select, rotate rows]
   - Directly. Be open and honest about what might improve my/my loved one’s health.
   - Gently. This may be hard for me/my loved one to hear.
   - Slowly. Don’t rush; I/my loved one may need some time to think about this.
   - Empathetically. I/my loved one would want to know that you are interested in how we feel.
   - Simply. I/my loved one don’t need all the details right now.
   - Clearly. The more details the better.
   - Other, please specify: [anchor, open-end]
   - N/A – I don’t ever want to talk to healthcare practitioners about weight management [anchor, exclusive]
   - I prefer not to answer [anchor, exclusive]
8. Have you or your loved one ever delayed or cancelled your own care due to the treatment you experienced related to weight? [multi-select]
   - Yes, I or my loved one has delayed care
   - Yes, I or my loved one has cancelled care
   - No [exclusive]
   - I prefer not to answer [exclusive]
9. Imagine your healthcare provider is talking with you about your, or a loved one’s weight. How would you feel about your healthcare provider using the words below? [rotate rows]

|  | Very positive | Somewhat positive | Neither positive nor negative | Somewhat negative | Very negative |
| --- | --- | --- | --- | --- | --- |
| BMI | o | o | o | o | o |
| Weight loss | o | o | o | o | o |
| Overweight | o | o | o | o | o |
| Obese/Obesity | o | o | o | o | o |
| Underweight | o | o | o | o | o |
| Thin | o | o | o | o | o |
| Diet | o | o | o | o | o |
| Healthy eating plan | o | o | o | o | o |
| Weight gain | o | o | o | o | o |
| Weight reduction | o | o | o | o | o |
| Exercise | o | o | o | o | o |
| Activity | o | o | o | o | o |

1. Who helps supports you in your health? [multi-select, rotate rows]
   - My partner/spouse
   - My parents
   - My siblings
   - My children
   - My friends
   - My healthcare provider
   - Members of my church or religious community
   - Other, please specify: [anchor, open-end]
   - N/A – I take care of my own health [anchor, exclusive]

**DEMOGRAPHICS**

1. For each statement about a household’s food situation, please mark whether the statement was often true, sometimes true or never true for your household in the last 12 months.

|  | Often true | Sometimes true | Never true | Prefer not to say |
| --- | --- | --- | --- | --- |
| We worried whether our food would run out before we got money to buy more. | o | o | o | o |
| The food that we bought just didn’t last, and we didn’t have money to get more. | o | o | o | o |

1. Anything else you would like us to know about healthcare related to weight or size? [Optional essay]
2. If you would like to remain involved with enhancing OHSU’s management of obesity on a system-wide basis, please include your email or a phone number as a point of contact below. [optional]
   - First Name: [open-ended]
   - Last Name: [open-ended]
   - Email Address: [open-ended]
   - Phone number: [open-ended]
