# Supplementary material for: Patient perceived weight stigma and patient-centered language use preferences: A cross-sectional mixed methods analysis conducted in a large academic medical center
Source: PLoS One. 2025 Feb 10;20(2):e0314269. doi: 10.1371/journal.pone.0314269 (PMC11809864; doi:10.1371/journal.pone.0314269)
Supplement: Attachment — Submitted filename: PLOS ONE COMMENTS TO THE AUTHORS.pdf [file pone.0314269.s002.pdf]

Dear Authors,

Thank you for the opportunity to review your manuscript. While your research study is critically important in informing practice and health research broadly, I have identified several areas of concern that need addressing.

(i) Manuscript Formatting Guidelines:

All headings must be written in sentence case (capitalize only the first word of the heading, the first word of the subheading, and any proper nouns).

1. Abstracts:

Background

[Page 2, line 44]: "Given this healthcare system challenge, patient-specific context is needed to optimize weight management with an emphasis on reducing health care-associated weight stigma."

The phrase "Given this healthcare system challenge" is somewhat vague and could be clarified. Instead, you could say "Due to the prevalence of obesity and its impact on healthcare……….".

[Page 2, line 46]: "We sought to identify attitudes, behaviors, and institutional systems or structures that hinder and facilitate patients' weight management to inform future systematic improvements".

The sentence is long and could be more concise and specific about the objectives of the study. This could be phrased as "Our study aimed to explore patient attitudes, behaviours, and institutional factors that affect weight management and identify barriers and facilitators for improving care systematically".

Material and methods.

[Page 2, line 48]: "A cross-sectional survey of individuals opted-in to complete patient experience surveys after receiving care"- The phrasing is somewhat unclear.

Authors may need to clarify this sentence as follows: “The study utilised a cross-sectional survey among individuals who opted to complete patient experience surveys after receiving care……….”.

Conclusion.

[Page 2, line 61 - 65]: While this study provides practical implications for healthcare systems aiming to improve weight management and reduce stigma, it is essential for the authors to articulate a novel scholarly contribution of their findings in the existing body of knowledge in this field.

2. Introduction:

[Page 3, line 70 - 76]: “The management of overweight and obesity as a chronic disease is complicated by the multifactorial impact that individual factors (e.g., genetic and co-occurring medical conditions), psychological factors (e.g., weight stigma and behavioral), and societal factors (e.g., social drivers of health) have upon individuals with overweight or obesity.[2-5] Given this concerning trend and treatment complexity, national and international organizations have recommended a multi-level, multi-modal approach with healthcare institutions integrating context-specific clinical and community-level interventions through a chronic care model.[3, 6, 7]”

[Page 3, line 70 - 73]: The first sentence is quite long and could be precise by breaking down into more digestible parts. Suggested line should be phrased succinctly as follows: “Managing overweight and obesity as chronic diseases is challenging due to their multifactorial nature, influenced by individual (e.g., genetic predisposition, co-existing medical conditions), psychological (e.g., weight stigma, behavioral), and societal factors (e.g., social determinants of health). [2-5]”.

[Page 3, line 73 - 76]: The second sentence should specific which “national and international organizations have made these recommendations” and provide clarification in terms of what is “context-specific clinical and community-level

interventions through a chronic care model" to clarify for audience (readers) who may not be familiar with these terms/phrases.

[Page 3, line 78 - 85]: "Studies assessing patient and provider communication when discussing weight management have revealed that many patients with obesity experience explicit and implicit stigma when interacting with healthcare providers and systems.[8] Moreover, a recent review modeled how negative provider attitudes and behaviors, such as enacted stigma, threatening environmental cues, and stereotypical behaviors towards patients with obesity can strain the patient-clinician relationship, resulting in adverse patient outcomes (e.g., delayed/avoidant care seeking behaviors, decreased adherence to physician recommendations, and lower likelihood of weight loss).[9, 10]"

Authors may need to provide examples supporting this assertion "negative provider attitudes and behaviors," to specify instances of these behaviors (e.g., the use of stigmatizing language, focusing solely on weight without considering broader health issues).

Furthermore, consider citing specific studies or reports that illustrate patient experiences of stigma rather than general broad statements (e.g., provide details from qualitative studies with the data that describe patient narratives).

[Page 4, line 102 - 106]: "To assess this, we conducted a cross-sectional survey with an existing institutional group of patients through our organization's contracted survey vendor (NRC Health). The primary aim of this survey was to understand patient experiences at our institution, including their preferences for communication about obesity care, to inform future health system improvement."

Authors should provide a clear rationale grounded in existing literature or studies on why understanding patient perceptions of weight stigma and preferences for language use is crucial for healthcare outcomes. For instance, cite most recent studies rather than official websites that link weight stigma to adverse health behaviors or outcomes (e.g., delayed care, non-adherence to treatment).

Likewise, authors ought to specify the gaps in current literature or evidence that the study aims to address. This could include gaps in understanding how different communication approaches affect patient engagement in weight management.

[Page 4, line 96 - 102]: "The institutional guideline development process aimed to improve the management of people living with overweight and obesity through developing guidelines, standards, and policies that promote optimal patient health. A multi-disciplinary team of experts from across the institution convened for 3 years to review the relevant evidence base and develop guidelines appropriate for this institutional context. During this process, we identified the importance of including how patients with overweight or obesity in our health system perceived their prior encounters for weight management care, particularly when discussing weight management with their primary care providers."

Provide empirical evidence or theoretical frameworks that support the significance of patient-centred language in healthcare interactions, particularly in the context of obesity management. This could include references to primary studies. The study could draw upon frameworks like the Patient-Centered Medical Home (PCMH) model which focuses comprehensive and coordinated care that is centred on patient preferences, needs, and values. Read: https://doi.org/10.1186/s13643-022-02132-x and consider utilizing patient-centred approaches in obesity management, which take into account patient perceptions and preferences. Read: https://doi.org/10.1016/j.jand.2022.01.004 and https://doi.org/10.1159/000496183

3. Material and Methods:

[Page 5, line 135 - 136]: "The survey was also translated into Spanish for primary care clinics that primarily serve Spanish-speaking patients and distributed online through specific clinic partnerships."

Authors must specify who performed the initial translation into Spanish and the qualifications of the translators (e.g., bilingual experts in healthcare communication)

and describe the back-translation process, where the translated survey was translated back into English to verify accuracy and consistency.

Pilot testing is crucial to identify and resolve any issues with survey comprehension, clarity, and appropriateness before full-scale implementation. Provide details if applicable on how the pilot testing was conducted. Furthermore, authors should provide preliminary or baseline results from the validation process to demonstrate the reliability and validity of survey items.

4. Results:

[Page 7, line 174 - 175]: “Only 2.3% had underweight, 30.4% had healthy weights, 31.5% had overweight, and 35.9% had obesity.”

[Page 10, line 214 - 215]: “Only 1.3% of males….”

To ensure consistency authors should make certain that all percentages are placed within parentheses for clarity.

5. Discussion:

[Page 22, line 317 - 318]: “However, females with class 2 and 3 obesity preferred a more indirect approach.”

The term “indirect approach” is vague and needs clarification or specific examples to support this assertion.

[Page 24, line 347 - 348]: “There are several limitations of our improvement project, which focused on the adult patient population served by a single institution.”

“Improvement project” is an unclear term in this context. It should be replaced with a more specific description of the study, such as “Research project”.

[Page 21, line 290 - 294]: “Notably, previous research has indicated that patients are not always in agreement with providers about whether they have discussed weight management in a visit.[17] Nevertheless, a second strong recommendation is for providers, with permission, to increasingly include the topic of weight management in their care...”

Authors need to establish a smoother transition between the statement about disagreement and the recommendation for providers.

Consider restructuring this section as follows: “Previous research suggests that patients and providers may not always agree on whether weight management has been discussed during visits. [17] Despite this, it is strongly recommended that providers……….”

[Page 22, line 312 - 315]: “Our finding that respondents preferred direct, clear, and empathetic styles of communication, across all BMI categories is similar to ***Koball, et al. (2018)*** who reported that patients preferred “very direct/straightforward” communication.[23]”

Authors must ensure that citations are consistently and accurately formatted using one citation style for clarity and credibility.

[Page 22, line 312 - 315]: This part of a sentence needs clarity “Our finding that respondents preferred direct…….” It could be rewritten for clarity: “Our finding indicates that respondents preferred direct…….”.

[Page 22, line 297]: This phrase needs revision “Qualitative feedback….” It could be rephrased as: “Qualitative findings…….”.

Thank you.
