# Supplementary material for: Patient perceived weight stigma and patient-centered language use preferences: A cross-sectional mixed methods analysis conducted in a large academic medical center
Source: PLoS One. 2025 Feb 10;20(2):e0314269. doi: 10.1371/journal.pone.0314269 (PMC11809864; doi:10.1371/journal.pone.0314269)
Supplement: Attachment — Submitted filename: Response to Reviewers.RMK.docx [file pone.0314269.s003.docx]

Dear PLOS ONE Editorial Team and Reviewers,

Thank you for your thorough review and opportunity to resubmit our manuscript entitled: “Patient perceived weight stigma and patient-centered language use preferences: a cross-sectional mixed methods quality improvement project in a large academic medical center.” We have worked to incorporate your feedback into our manuscript to the best of our ability.

Please see the below direct responses to all reviewer feedback. All reviewer feedback has been pasted below to facilitate ease of identification of our responses. We look forward to hearing from you soon regarding our revised manuscript.

Kind Regards,

Ryan M. Kane, MD, MPH

Medical Instructor and National Clinician Scholar

Division of General Internal Medicine, Department of Medicine

Duke University School of Medicine

Duke Clinical and Translational Science Institute

710 W Main St., Durham, NC 27701

[ryan.kane@duke.edu](mailto:ryan.kane@duke.edu)

i Manuscript Formatting Guidelines:

All headings must be written in sentence case (capitalize only the first word of the heading, the first word of the subheading, and any proper nouns).

>>Response:

Thank you for this formatting insight. We have updated all manuscript headings accordingly.

1. **Abstracts:**

**Background**

**[Page 2, line 44]:** “Given this healthcare system challenge, patient-specific context is needed to optimize weight management with an emphasis on reducing health care-associated weight stigma.”

The phrase "Given this healthcare system challenge" is somewhat vague and could be clarified. Instead, you could say “Due to the prevalence of obesity and its impact on healthcare……….”.

>>Response:

Thank you for this suggestion, we have updated this language based on your suggested phrasing to improve specificity: “Due to the rising prevalence of obesity and its impact on healthcare, patient-specific…”

**[Page 2, line 46]:** “We sought to identify attitudes, behaviors, and institutional systems or structures that hinder and facilitate patients’ weight management to inform future systematic improvements”.

The sentence is long and could be more concise and specific about the objectives of the study. This could be phrased as “Our study aimed to explore patient attitudes, behaviours, and institutional factors that affect weight management and identify barriers and facilitators for improving care systematically”.

>>Response:

Thank you for this suggestion, we have updated this language to enhance clarity based on your suggested phrasing: “Our study aimed to explore patient attitudes, behaviors, and institutional factors that affect weight management to identify barriers and facilitators for systematically improving care.”

**Material and methods.**

**[Page 2, line 48]:** “A cross-sectional survey of individuals opted-in to complete patient experience surveys after receiving care”- The phrasing is somewhat unclear.

Authors may need to clarify this sentence as follows: “The study utilised a cross-sectional survey among individuals who opted to complete patient experience surveys after receiving care……….”.

>>Response:

Thank you for this suggestion, we have updated this language to enhance clarity based on your suggested phrasing to “This study used a cross-sectional survey among individuals who previously opted-in to complete patient experience surveys after receiving care in a large U.S. academic medical center.”

**Conclusion.**

**[Page 2, line 61 - 65]:** While this study provides practical implications for healthcare systems aiming to improve weight management and reduce stigma, it is essential for the authors to articulate a novel scholarly contribution of their findings in the existing body of knowledge in this field.

>>Response:

We appreciate the need to clarify our study’s unique contribution to the field whilst staying within the 300-word count maximum of the abstract. We believe our new abstract conclusion addresses this well [line 61-65]:

“Findings from our large single institution cohort expand on the existing weight stigma literature by identifying patient language preferences and healthcare experiences according to patient weight class and sex. Given the potential impact of understanding context-specific patient language use preferences to reduce weight stigma, we recommend other healthcare systems use a similar process to develop institutional plans that address weight stigma as part of a coordinated systems approach for weight management.”

2. **Introduction:**

**[Page 3, line 70 - 76]:** “The management of overweight and obesity as a chronic disease is complicated by the multifactorial impact that individual factors (e.g., genetic and co-occurring medical conditions), psychological factors (e.g., weight stigma and behavioral), and societal factors (e.g., social drivers of health) have upon individuals with overweight or obesity.[2-5] Given this concerning trend and treatment complexity, national and international organizations have recommended a multi-level, multi-modal approach with healthcare institutions integrating context-specific clinical and community-level interventions through a chronic care model.[3, 6, 7]”

**[Page 3, line 70 - 73]:** The first sentence is quite long and could be precise by breaking down into more digestible parts. Suggested line should be phrased succinctly as follows: “Managing overweight and obesity as chronic diseases is challenging due to their multifactorial nature, influenced by individual (e.g., genetic predisposition, co-existing medical conditions), psychological (e.g., weight stigma, behavioral), and societal factors (e.g., social determinants of health). [2-5]”.

>>Response:

Thank you for this suggested modification. We have made this more succinct, as follows: “Managing obesity as chronic disease is challenging due to its multifactorial nature — influenced by individual factors (e.g., genetic and co-occurring medical conditions), psychological factors (e.g., weight stigma and behavioral), and societal factors (e.g., social drivers of health).”

**[Page 3, line 73 - 76]:** The second sentence should specific which “national and international organizations have made these recommendations” and provide clarification in terms of what is “context-specific clinical and community-level

interventions through a chronic care model” to clarify for audience (readers) who may not be familiar with these terms/phrases.

>>Response:

Thank you for offering insights into points to clarify here. I have added additional detail on the institutions and the chronic care model. “Given this concerning trend and treatment complexity, the National Academies of Sciences, Engineering, and Medicine and World Health Organization have recommended multi-level, multi-modal approaches for healthcare institutions to work within their individual clinical and community environments to enhance the prevention of and treatment for obesity, using a chronic care model.[3, 6, 7] The chronic care model highlights critical elements for health care systems to enhance the provision of high-quality chronic disease care across patient, health system, and community spheres of influence.[8]”

**[Page 3, line 78 - 85]:** “Studies assessing patient and provider communication when discussing weight management have revealed that many patients with obesity experience explicit and implicit stigma when interacting with healthcare providers and systems.[8] Moreover, a recent review modeled how negative provider attitudes and behaviors, such as enacted stigma, threatening environmental cues, and stereotypical behaviors towards patients with obesity can strain the patient-clinician relationship, resulting in adverse patient outcomes (e.g., delayed/avoidant care seeking behaviors, decreased adherence to physician recommendations, and lower likelihood of weight loss).[9, 10]”

Authors may need to provide examples supporting this assertion “negative provider attitudes and behaviors,” to specify instances of these behaviors (e.g., the use of stigmatizing language, focusing solely on weight without considering broader health issues).

Furthermore, consider citing specific studies or reports that illustrate patient experiences of stigma rather than general broad statements (e.g., provide details from qualitative studies with the data that describe patient narratives).

>>Response:

Due to the amount of information in this sentence, I broke it up for the sake of clarity and added additional references from qualitative studies to support these statements:

“Moreover, a recent review modeled how negative provider attitudes and behaviors can strain the patient-clinician relationship, resulting in adverse patient outcomes (e.g. delayed/avoidant care seeking behaviors, decreased adherence to physician recommendations, and lower likelihood of weight loss).[10, 11] These negative provider attitudes and behaviors include (1) enacted stigma (behaviors resulting from negative attitudes like decreased patient-centered communication due to providers’ belief that patients with obesity are less likely to adhere to treatments), (2) threatening environmental cues (clinical spaces and equipment that are not accommodating to patients of all sizes), and stereotypical behaviors (providers demonstrating less respect towards patients with obesity due to perceptions of laziness).[10] Review of qualitative analyses identified related emergent themes surrounding (1) verbal/non-verbal communication of stigma, (2) the impact of weight stigma upon care provision, and (3) weight stigma’s impact upon systemic barriers to care.[12]”

**[Page 4, line 102 - 106]:** “To assess this, we conducted a cross-sectional survey with an existing institutional group of patients through our organization’s contracted survey vendor (NRC Health). The primary aim of this survey was to understand patient experiences at our institution, including their preferences for communication about obesity care, to inform future health system improvement.”

Authors should provide a clear rationale grounded in existing literature or studies on why understanding patient perceptions of weight stigma and preferences for language use is crucial for healthcare outcomes. For instance, cite most recent studies rather than official websites that link weight stigma to adverse health behaviors or outcomes (e.g., delayed care, non-adherence to treatment).

Likewise, authors ought to specify the gaps in current literature or evidence that the study aims to address. This could include gaps in understanding how different communication approaches affect patient engagement in weight management.

>>Response:

Thank you for this recommendation. We have bolstered the literature base in this paragraph and provided additional context regarding health system gaps. “Due to the literature on weight stigma’s deleterious effects for patient engagement in preventive care, healthcare avoidance, biased physician decision making, poor patient-provider communication, and increased primary care switching; we sought to understand how patients with overweight or obesity in our health system perceived their prior care experiences.[10, 15-18] Though prior literature demonstrated weight stigma-focused analyses of specific cohorts, we were unaware of prior health systems who had published a similar institutional approach to better understand the direct language use preferences and care experiences of their own patient population.”

**[Page 4, line 96 - 102]:** “The institutional guideline development process aimed to improve the management of people living with overweight and obesity through developing guidelines, standards, and policies that promote optimal patient health. A multi-disciplinary team of experts from across the institution convened for 3 years to review the relevant evidence base and develop guidelines appropriate for this institutional context. During this process, we identified the importance of including how patients with overweight or obesity in our health system perceived their prior encounters for weight management care, particularly when discussing weight management with their primary care providers.”

Provide empirical evidence or theoretical frameworks that support the significance of patient-centred language in healthcare interactions, particularly in the context of obesity management. This could include references to primary studies. The study could draw upon frameworks like the Patient-Centered Medical Home (PCMH) model which focuses comprehensive and coordinated care that is centred on patient preferences, needs, and values. Read: https://doi.org/10.1186/s13643-022-02132-x and consider utilizing patient-centred approaches in obesity management, which take into account patient perceptions and preferences. Read: https://doi.org/10.1016/j.jand.2022.01.004 and https://doi.org/10.1159/000496183

>>Response:

Thank you for these helpful insights. I have reviewed your citations and included highlighted the importance of patient-centered communication, motivational interviewing, and evidence-based obesity treatment grounded in the literature (including the NAM framework by Dietz, et al.).

“The goal of this is to ensure evidence-based, patient-centered obesity care is available to all patients in our health system to reduce the experience of weight stigma, improve health outcomes, reduce costs, and limit patient harm. Much of this starts with provider communication and the use of patient-centered language or motivational interviewing to compassionately engage patients in a conversation about their weight.[15-17]”

3. **Material and Methods:**

**[Page 5, line 135 - 136]:** “The survey was also translated into Spanish for primary care clinics that primarily serve Spanish-speaking patients and distributed online through specific clinic partnerships.”

Authors must specify who performed the initial translation into Spanish and the qualifications of the translators (e.g., bilingual experts in healthcare communication)

and describe the back-translation process, where the translated survey was translated back into English to verify accuracy and consistency.

Pilot testing is crucial to identify and resolve any issues with survey comprehension, clarity, and appropriateness before full-scale implementation. Provide details if applicable on how the pilot testing was conducted. Furthermore, authors should provide preliminary or baseline results from the validation process to demonstrate the reliability and validity of survey items.

>>Response:

Thank you for these comments. I have adjusted the text accordingly to respond to these comments:

“After the survey was developed by national survey experts from NRC Health, it was iteratively reviewed by the institutional weight management guideline subcommittee for survey development as well as the multi-stakeholder guideline development team to enhance face and content validity prior to its administration via email between September 29, 2021 and October 5, 2021.[23] The survey was also translated into Spanish by certified bilingual healthcare interpreters for primary care clinics”

Regarding requested information from “pilot testing” and “preliminary or baseline results from the validation process,” we have revised the limitations section to indicate that we did not conduct rigorous test-retest analyses to provide validity data, but we are confident that our iterative expert and stakeholder review process resulted in a survey that produced accurate results. Given the initial focus if this project as a quality improvement project and our use of a national survey vendor (NRC Health) contracted with our institution, pilot testing beyond face and content validity was not planned as part of NRC Health’s standard process when working with health systems on projects such as ours. We did previously acknowledge this limitation in the limitations section of our Discussion though have bolstered this section, as follows: “The overall survey had very few previously validated questions to assess patient preferences and was not pre-tested among a pilot population prior to full dissemination; though it was developed in partnership with a national third-party vendor and informed by iterative multi-disciplinary clinician expert meetings to establish face and content validity. Future partnerships between clinicians and national survey vendors contracted with health systems should consider pilot testing to enhance survey comprehension, clarity, and appropriateness prior to full dissemination.”

4. **Results:**

**[Page 7, line 174 - 175]:** “Only 2.3% had underweight, 30.4% had healthy weights, 31.5% had overweight, and 35.9% had obesity.”

**[Page 10, line 214 - 215]:** “Only 1.3% of males….”

To ensure consistency authors should make certain that all percentages are placed within parentheses for clarity.

>>Response:

Thank you for this suggestion. I have rearranged these sentences for clarity:

“Respondents were distributed across weight classes: underweight (2.3%), healthy weight (30.4%), overweight (31.5%), and obesity (35.9%).”

“Males (1.3%) rarely indicated…”

5. **Discussion:**

**[Page 22, line 317 - 318]:** “However, females with class 2 and 3 obesity preferred a more indirect approach.”

The term “indirect approach” is vague and needs clarification or specific examples to support this assertion.

>>Response:

We recognize that the use of “indirect” is somewhat vague here. This was in reference to Table 3’s “Female” respondents with class 2 and 3 obesity having a lower frequency for preference of a “Directly (Open & Honest)” response to the question: “How would you want your provider to talk to you about weight management” (p-value <0.005). This question did not have associated space to provide direct quotes that would expand on this, though I added in a related quote from our qualitative analysis. I have adjusted this section accordingly to enhance the statement’s clarity: “However, females with class 2 and 3 obesity less often preferred a direct approach (see Table 3), exemplified by one participant’s response: “My preference is for providers to approach in a friendly empathetic way with a gradual supportive approach…” *[Participant #OC2-212]* (“Empathetic Communication” theme).”

**[Page 24, line 347 - 348]:** “There are several limitations of our improvement project, which focused on the adult patient population served by a single institution.”

“Improvement project” is an unclear term in this context. It should be replaced with a more specific description of the study, such as “Research project”.

>>Response:

Thank you for this comment. The nature of our project began as a health system-wide quality improvement project to enhance guideline-based weight management care at our institution. This survey was a portion of this project that developed out of our desire to understand the experiences and preferences of our specific population. Thus, the design of the study we present was more in keeping with a quality improvement project, as described in the title, methods, and here in the discussion. For the sake of clarity, I have adjusted the sentence accordingly: “There are several limitations of this analysis…”

**[Page 21, line 290 - 294]:** “Notably, previous research has indicated that patients are not always in agreement with providers about whether they have discussed weight management in a visit.[17] Nevertheless, a second strong recommendation is for providers, with permission, to increasingly include the topic of weight management in their care...”

Authors need to establish a smoother transition between the statement about disagreement and the recommendation for providers.

Consider restructuring this section as follows: “Previous research suggests that patients and providers may not always agree on whether weight management has been discussed during visits. [17] Despite this, it is strongly recommended that providers……….”

>>Response:

Thank you for recognizing this transition issue. I appreciate your suggested text and have updated the sentence accordingly: “Previous research suggests that patients and providers may not always agree on whether weight management has been discussed during visits.[27] Despite this, it is strongly recommended for providers, with permission, to increasingly include the topic of weight management in their care…”

**[Page 22, line 312 - 315]:** “Our finding that respondents preferred direct, clear, and empathetic styles of communication, across all BMI categories is similar to ***Koball, et al. (2018)*** who reported that patients preferred “very direct/straightforward” communication.[23]”

Authors must ensure that citations are consistently and accurately formatted using one citation style for clarity and credibility.

>>Response:

Thank you for this input. We included study author names in text sparingly to highlight to the reader when we were talking about similar studies. We did use the same citation format per PLOS ONE’s preference, Vancouver, throughout. The in text notation for Koball was appropriately cited as “23” after the sentence. I had included dates in a few places to highlight the relatively recent nature of a couple of studies, given language preferences can evolve rapidly over short periods of time. That said, I have removed the date reference here and from another sentence that followed. This sentence has been updated accordingly: “Our findings (across all BMI categories) indicating that respondents preferred direct, clear, and empathetic styles of communication is similar to Koball, et al. who reported that patients preferred “very direct/straightforward” communication.[33]” Note that the citation number has changed due to my adding several more citations prior to this sentence.

**[Page 22, line 312 - 315]:** This part of a sentence needs clarity “Our finding that respondents preferred direct…….” It could be rewritten for clarity: “Our finding indicates that respondents preferred direct…….”.

>>Response:

Thank you for this suggestion. I have rewritten this sentence for clarity as follows: “Our findings (across all BMI categories) indicating that respondents preferred direct, clear, and empathetic styles of communication is similar to Koball, et al…”

**[Page 22, line 297]:** This phrase needs revision “Qualitative feedback….” It could be rephrased as: “Qualitative findings…….”.

>>Response:

Thank you for this correction. We have adjusted the phrase to “Qualitative findings…”

>> END OF LETTER <<
